# Non‐lethal sampling for the stable isotope analysis of the critically endangered European eel *Anguilla anguilla*: how fin and mucus compare to dorsal muscle

**DOI:** 10.1111/jfb.14992

**Published:** 2022-02-09

**Authors:** Rose M. Boardman, Adrian C. Pinder, Adam T. Piper, Catherine Gutmann Roberts, Rosalind M. Wright, J. Robert Britton

**Affiliations:** ^1^ Department of Life and Environmental Sciences, Faculty of Science and Technology Bournemouth University Poole UK; ^2^ Institute of Zoology Zoological Society of London London UK; ^3^ School of Geography, Earth and Environmental Science University of Plymouth Plymouth UK; ^4^ Environment Agency Feering UK

**Keywords:** Anguillid, non‐destructive sampling, Red List species, trophic ecology

## Abstract

Ecological studies on the critically endangered European eel *Anguilla anguilla* often incorporate stable isotope analysis that typically uses dorsal muscle sampled from euthanised eels. To minimise the lethal sampling of imperilled populations, fin tissue and/or epidermal mucus can provide non‐lethal alternatives to muscle. The results here indicate that δ^13^C and δ^15^N values of both eel fin and mucus are not significantly different from those of muscle and can be applied directly in comparative SI studies.

Natural chemical tags, such as stable isotopes, are widely used to investigate the spatial and trophic ecology of fishes (Trueman *et al*., 2012), with the stable isotope ratios of carbon and nitrogen (as δ^13^C and δ^15^N) commonly applied to trophic ecology and food web studies (Fry, 2006; Perkins *et al*., 2014). Applications include providing information on diet composition (Nolan *et al*., 2019), dietary shifts (Vander Zanden & Rasmussen, 1999), consumer trophic positions (Post, 2002) and foraging patterns (Cunjak *et al*., 2005).

The stable isotope analysis (SIA) of fishes is typically performed on samples of dorsal muscle (Pinnegar & Polunin, 1999), with sampling involving the euthanasia of individuals that are then dissected in the laboratory (Sanderson *et al*., 2009). Although muscle can also be sampled non‐lethally through the collection of a biopsy sample *via* a muscle plug, these samples can introduce bias in the SI data when used on smaller fish (Schielke & Post, 2010). As neither lethal sampling nor biased data are desirable, especially when working on threatened species, alternative tissues, such as fin and scale tissue, that can be collected non‐destructively are increasingly used (Hutchinson & Trueman, 2006; Nolan *et al*., 2019; Sanderson *et al*., 2009).

Although these alternative tissues can provide reliable substitutes for dorsal muscle in SI studies, their values often need correction factors to be applied if they are to be compared with dorsal muscle values from other studies (Kelly *et al*., 2006; Maitland & Rahel, 2021; Roberts *et al*., 2021). For example, values of δ^13^C of fin and scales tend to be enriched compared with those of muscle (Winter *et al*., 2019a, 2019b). SI values of muscle, fin and scales are also usually highly correlated within species, meaning their differences are highly predictable (Busst *et al*., 2015; Sanderson *et al*., 2009). In recent years, epidermal mucus has also provided a further reliable source of analytical material for studying fish SI, although when compared with other tissues, it tends to have a faster isotopic turnover rate (Winter *et al*., 2019b; Winter & Britton, 2021).

In recent decades, the European eel *Anguilla anguilla* L. 1758 has undergone rapid declines in recruitment and abundance across its range and, since 2008, has been assessed as Critically Endangered on the IUCN Red List of Threatened Species (Pike *et al*., 2020). SIA is frequently used in ecological studies of *A. anguilla*, where it has been used to identify their trophic ecology across salinity gradients (Harrod *et al*., 2005), their dietary differences in relation to head morphology (Cucherousset *et al*., 2011) and aspects of parasite infection (Pegg *et al*., 2015). Although fin tissue is commonly used in eel SI studies (*e.g*., Cucherousset *et al*., 2011; Musseau *et al*., 2015), many studies still use dorsal muscle, with samples collected from euthanised fish (*e.g*., Capoccioni *et al*., 2021; Parzanini *et al*., 2021).

To date, determining the relationships of SI values between different fish tissues and how these tissues can be applied in non‐lethal sampling programmes has mainly focused on species of the Salmonidae and Cyprinidae families (*e.g*., Busst *et al*., 2015; Church *et al*., 2009). Despite their imperilled status, no similar relationships are currently available for Anguillids. Consequently, the aim here was to determine how fin and mucus samples could be used to replace dorsal muscle samples in the SIA of eels. This was completed by sampling juvenile *A. anguilla* (*n* = 43) from a side‐stream located on the lower reaches of the River Frome (51° 20′ 21″N; 2° 17′ 44″W; *n* = 19) and from an elver pass on the River Piddle (50° 40′ 59”N; −2°03′60” W; *n* = 24), Southern England, in April 2021 (Table 1). Note these two rivers drain into the same location within Poole Harbour and have similar physical and chemical characteristics (Humphreys & May, 2005). The River Frome was sampled by back‐mounted electric fishing (SmithRoot LR24) and the River Piddle using a trap operated over 24 h periods on an existing elver pass.

**TABLE 1:** Sample size and mean and range as minimum (“min”) and maximum (“max”) of total length (“length”), δ^13^C (following mathematical lipid normalisation) and δ^15^N for fin, dorsal muscle (“muscle”) and mucus of the samples of *Anguilla anguilla*

Tissue comparison	*n*	Mean length ± 95% c.i. (min, max) (mm)	Tissue	Mean δ^13^C ± 95% c.i. (min, max) (‰)	Mean δ^15^N ± 95% c.i. (min, max) (‰)
Muscle/mucus	43	116 ± 17 (67, 320)	Muscle	−29.1 ± 1.8 (−33.5, −20.9)	11.0 ± 1.1 (5.8, 13.2)
			Mucus	−29.0 ± 1.7 (−32.1–20.8)	11.3 ± 1.2 (5.3, 13.7)
Muscle/fin	6	232 ± 49 (147, 320)	Muscle	−29.2 ± 1.5 (−31.6, −26.6)	12.3 ± 0.8 (10.4, 13.2)
			Fin	−29.1 ± 1.4 (−31.6, −26.8)	12.6 ± 0.7 (11.0, 13.5)

A sub‐sample of eels from both locations was euthanised (anaesthetic overdose, MS‐222), with individual eels placed into plastic sample bags and taken to the laboratory. There, each eel was measured [total length (TL), nearest millimetre] before a sample of dorsal muscle was excised from all individuals, and a fin sample was taken from all those >146 mm TL (fin tissue was not collected from eels <146 mm TL due to the limited fin tissue available on these individuals). A sample of epidermal mucus was then collected using a single‐use, sterile cover slip and running it lightly along the length of one side of the eel, with this capturing sufficient mucus on the cover slip for SIA, and with no further treatment of this mucus sample other than its transfer to an individual sample tube (Winter *et al*., 2019a, 2019b; Winter & Britton, 2021).

All muscle, fin and mucus samples were then dried to constant weight (60°C for 48 h), before being bulk analysed for δ^13^C and δ^15^N in a Thermo Delta V isotope ratio mass spectrometer (Thermo Scientific, Waltham, MA, USA) interfaced to a NC2500 elemental analyser (CE Elantach Inc., Lake‐ wood, NJ, USA). Analytical precision of the δ^13^C and δ^15^N sample runs was estimated against an internal standard sample of an animal (deer) material every 10 samples, with the overall standard deviation estimated at 0.08 and 0.04 ‰ respectively.

The C:N ratios of the samples varied according to tissue type, with the greatest range in dorsal muscle [3.40 to 5.01; mean (± 95% c.i.) 3.89 ± 0.28] and then fin (3.64 to 4.89; mean 4.12 ± 0.36), whereas mucus C:N ratios were comparatively low (3.56 to 3.84; mean 3.67 ± 0.06). Post *et al*. (2007) reported strong relationships between lipid content of tissues and both C:N ratios and δ^13^C, and suggested that lipid normalisation is important when lipid content is variable among consumer species. Nonetheless, relatively high and variable C:N ratios in fin tissues can be from the complex matrix of epidermal tissue and fin rays present in the analysed material (Hayden *et al*., 2015). Correspondingly, although the δ^13^C data were mathematically lipid normalised for further analyses, these analyses were also completed using the non‐normalised data and are provided in Supporting Information Tables TABLE S1 and TABLE S2 and Figure FIGURE S1). The δ^13^C values were then mathematically normalised for lipid using the equation of Kiljunen *et al*. (2006); all reported analyses on δ^13^C hereafter use these lipid normalised values.

The distributions of δ^13^C and δ^15^N were non‐normal (Shapiro–Wilk test, *P* < 0.05), and so differences in the SI values between muscle and fin, and muscle and mucus, were tested in paired Wilcoxon tests. These revealed that differences in SI values between these tissues were not significant (muscle *vs*. mucus: δ^13^C: *P* = 0.94, δ^15^N, *P* = 0.22; muscle *vs*. fin: δ^13^C: *P* = 0.87, δ^15^N, *P* = 0.58). When tested in linear regression, these relationships were all highly significant (*P* < 0.01; Table 2), with the 95% confidence limits of these regression relationships all overlapping the line of equality, suggesting differences were not significant (Figure 1). Nonetheless, the extent of overlap in confidence limits for the relationship of muscle *vs*. fin for δ^15^N was relatively minor, with a general pattern of enriched values of δ^15^N in the fin tissues (Figure 1d). A correction factor (*CF*) of the difference in the mean values of these tissues can thus be considered for converting fin δ^15^N to muscle δ^15^N values (*CF* = −0.33; Table 2).

**TABLE 2:** Linear regression statistics for the relationship between muscle stable isotope values [as δ^13^C (lipid normalised) and δ^15^N] and those of fin and mucus for *Anguilla anguilla*

Stable isotope	Tissue	*n*	Slope	Intercept	F	R^2^	*P*
δ^13^C	Fin	6	−1.90	0.93	106.2	0.96	<0.001
	Mucus	43	−1.10	0.96	529.8	0.92	<0.001
δ^15^N	Fin	6	1.78	0.88	106.5	0.95	<0.001
	Mucus	43	0.29	0.99	132.9	0.76	<0.001

**FIGURE 1:**
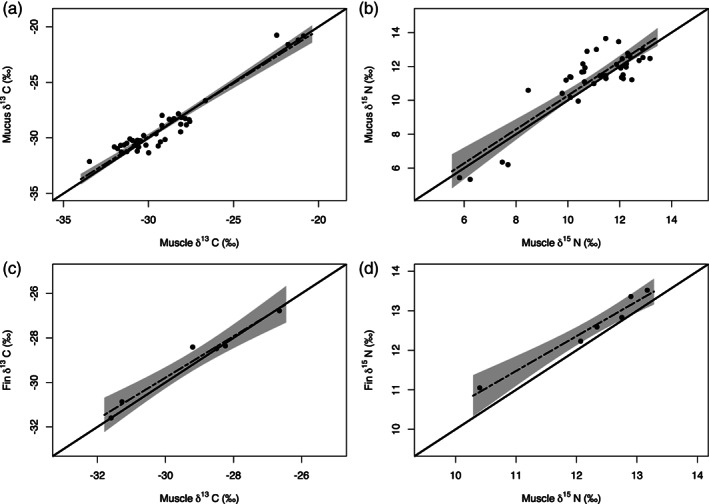
Relationships between lipid normalised δ^13^C (left) and δ^15^N (right) isotope signatures of muscle and mucus (a–b), and fin (c–d) in *Anguilla anguilla*. Bold lines indicate the line of equality, dashed lines represent the significant relationship of the variables according to linear regression (*P* < 0.01) and grey shading represents the 95% confidence limits of these linear relationships

These results demonstrate that epidermal mucus samples provide reliable and non‐lethal alternatives to the use of dorsal muscle and provide preliminary findings that fin could also be used as a non‐lethal alternative in larger individuals in SIA studies of *A. anguilla*. Furthermore, SI data can be compared directly between mucus and muscle without the requirement for correction factors to be applied. This is in contrast to most other fish species, such as Northern pike *Esox lucius* (Winter *et al*., 2019a) and common carp *Cyprinus carpio*, where the use of correction factors is often required due to predictable patterns in SI enrichment between muscle, fin and scales, with mucus samples often being depleted in their SI values (*e.g*., Winter *et al*., 2019b, Winter & Britton, 2021). Although it is recommended that a correction factor may be required when converting fin δ^15^N to muscle δ^15^N values, the small sample size (*n* = 6) means this should be used with caution. The sample size of muscle *vs*. fin tissues was limited to six eels here to minimise the number of larger individuals (>146 mm) that were euthanised. Although larger sample sizes of smaller individuals were used for testing muscle *vs*. mucus data, the abundance of these smaller eels remained relatively high in samples collected at both sites in subsequent weeks. For example, high numbers (>900) of elvers and yellow eels were captured in 24 h samples recorded from the elver trap on the River Piddle throughout May and June (the authors, unpubl. data). These trends suggest that the lethal sampling of these smaller eels conducted for this study did not impact their local abundances.

If mucus is to be used in future eel SI studies, then its faster SI turnover rate compared with both muscle and fin needs to be considered in both sampling design and evaluation (Winter *et al*., 2019b; Winter & Britton, 2021). Here, sampling was completed in April, based on *a priori* assumptions that the eel tissues were still in isotopic equilibrium with their long‐term diets. Therefore, glass eel/elvers that had only just entered fresh water were sampled, as these would provide individuals whose tissue isotope values would be largely marine based. Larger eels that had already settled in fresh water were also sampled as these would provide individuals whose tissue isotope values would still be in isotopic equilibrium with their diet from the previous summer/autumn periods, given the inactivity of eels in fresh water in cooler temperatures with minimal somatic body growth until water temperatures exceed 16°C (Vaughan *et al*., 2021). Thus, the SI relationships between the sampled tissues were considered as reflecting their actual patterns, with minimal influence of recent dietary changes affecting these. Nevertheless, knowledge on the isotopic turnover rates of δ^13^C and δ^15^N in Anguillid tissues remains highly limited and so it is recommended that these knowledge gaps are addressed if the full benefits of using non‐lethal tissue sampling in future SIA studies are to be realised.

To date, approximately half of all other Anguillid eel species are listed as vulnerable, endangered or critically endangered on the IUCN Red List (Itakura *et al*., 2019). As the SIA continues to be applied to studies on the ecology and conservation of these threatened species, the application of non‐lethal sampling *via* fin and/or mucus is thus encouraged wherever possible to avoid impacting their populations any further.

## CONFLICTS OF INTEREST

The authors declare that they are not aware of any competing interests.

## AUTHOR CONTRIBUTIONS

All authors were involved in the conceptualisation of the study, and in writing and editing the manuscript. R.M.B., J.R.B. and A.C.P. completed all sampling, and R.M.B. completed all data analyses and evaluation.

## ETHICAL STATEMENT

The study was completed following the gaining of all relevant ethical and legislative approvals (UK Home Office Project Licence P47216841; Environment Agency permit reference EP/EW027‐C‐042/19919/01).

## Supporting information


**FIGURE S1** Relationships between non‐corrected δ^13^C (left) and δ^15^N (right) isotope signatures of muscle and mucus (A–B), and fin (C–D) in *Anguilla anguilla*. Bold lines indicate the line of equality, dashed lines represent the significant relationship of the variables according to linear regression (*P* < 0.01) and grey shading represents the 95% confidence limits of these linear relationships.

**TABLE S1** Sample size and mean and range (as minimum (“min”) and maximum (“max”) of total length (“length”), δ^13^C (non‐corrected) and δ^15^N for fin, dorsal muscle (“muscle”) and mucus of the samples of *Anguilla anguilla*

**TABLE S2** Linear regression statistics for the relationship between muscle stable isotope values [as δ^13^C (non‐corrected) and δ^15^N] and those of fin and mucus for *Anguilla anguilla*.
