# Supplementary material for: Non‐lethal sampling for the stable isotope analysis of the critically endangered European eel Anguilla anguilla: how fin and mucus compare to dorsal muscle
Source: J Fish Biol. 2022 Feb 9;100(3):847–51. doi: 10.1111/jfb.14992 (PMC9303185; doi:10.1111/jfb.14992)
Supplement: Supplementary file 2 — TABLE S1 Sample size and mean and range (as minimum (“min”) and maximum (“max”) of total length (“length”), δ13C (non‐corrected) and δ15N for fin, dorsal muscle (“muscle”) and mucus of the samples of Anguilla anguilla [file JFB-100-847-s003.docx]

| Tissue comparison | n | Mean length ± 95 % CI  (min, max) (mm) | Tissue | Mean δ^13^C ± 95 % CI  (min, max) (‰) |
| --- | --- | --- | --- | --- |
| Muscle/ mucus | 43 | 116 ± 17  (67, 320) | Muscle | -30.1 ± 0.9  ( -35.3, -21.9) |
|  |  |  | Mucus | -29.9 ± 0.9  ( -33.1, -21.5) |
| Muscle/ fin | 6 | 232 ± 49  (147, 320) | Muscle | -30.4 ± 2.2  (-32.3, -27.3) |
|  |  |  | Fin | -30.3 ± 1.8  ( -32.7, -27.9) |

**Table S1:** Sample size and mean and range (as minimum (‘min’) and maximum (‘max’) of total length (‘length’), δ^13^C (non-corrected) and δ^15^N for fin, dorsal muscle (‘muscle’), and mucus of the samples of *Anguilla anguilla*
