# Supplementary material for: Non‐lethal sampling for the stable isotope analysis of the critically endangered European eel Anguilla anguilla: how fin and mucus compare to dorsal muscle
Source: J Fish Biol. 2022 Feb 9;100(3):847–51. doi: 10.1111/jfb.14992 (PMC9303185; doi:10.1111/jfb.14992)
Supplement: Supplementary file 3 — TABLE S2 Linear regression statistics for the relationship between muscle stable isotope values [as δ13C (non‐corrected) and δ15N] and those of fin and mucus for Anguilla anguilla. [file JFB-100-847-s001.docx]

**Table S2:** Linear regression statistics for the relationship between muscle stable isotope values (as δ^13^C (non-corrected) and δ^15^N) and those of fin and mucus for *Anguilla anguilla.*

| Stable isotope | Tissue | n | slope | intercept | F | R­^2^ | P |
| --- | --- | --- | --- | --- | --- | --- | --- |
| δ^13^C | Fin | 6 | -9.17 | 0.70 | 10.01 | 0.71 | < 0.03 |
|  | Mucus | 43 | -2.24 | 0.92 | 329.9 | 0.89 | < 0.001 |
| δ^15^N | Fin | 6 | 1.78 | 0.88 | 106.5 | 0.95 | < 0.001 |
|  | Mucus | 43 | 0.29 | 0.99 | 132.9 | 0.76 | < 0.001 |
