# Supplementary material for: Non‐lethal sampling for the stable isotope analysis of the critically endangered European eel Anguilla anguilla: how fin and mucus compare to dorsal muscle
Source: J Fish Biol. 2022 Feb 9;100(3):847–51. doi: 10.1111/jfb.14992 (PMC9303185; doi:10.1111/jfb.14992)
Supplement: Supplementary file 3 [file JFB-100-847-s002.docx]

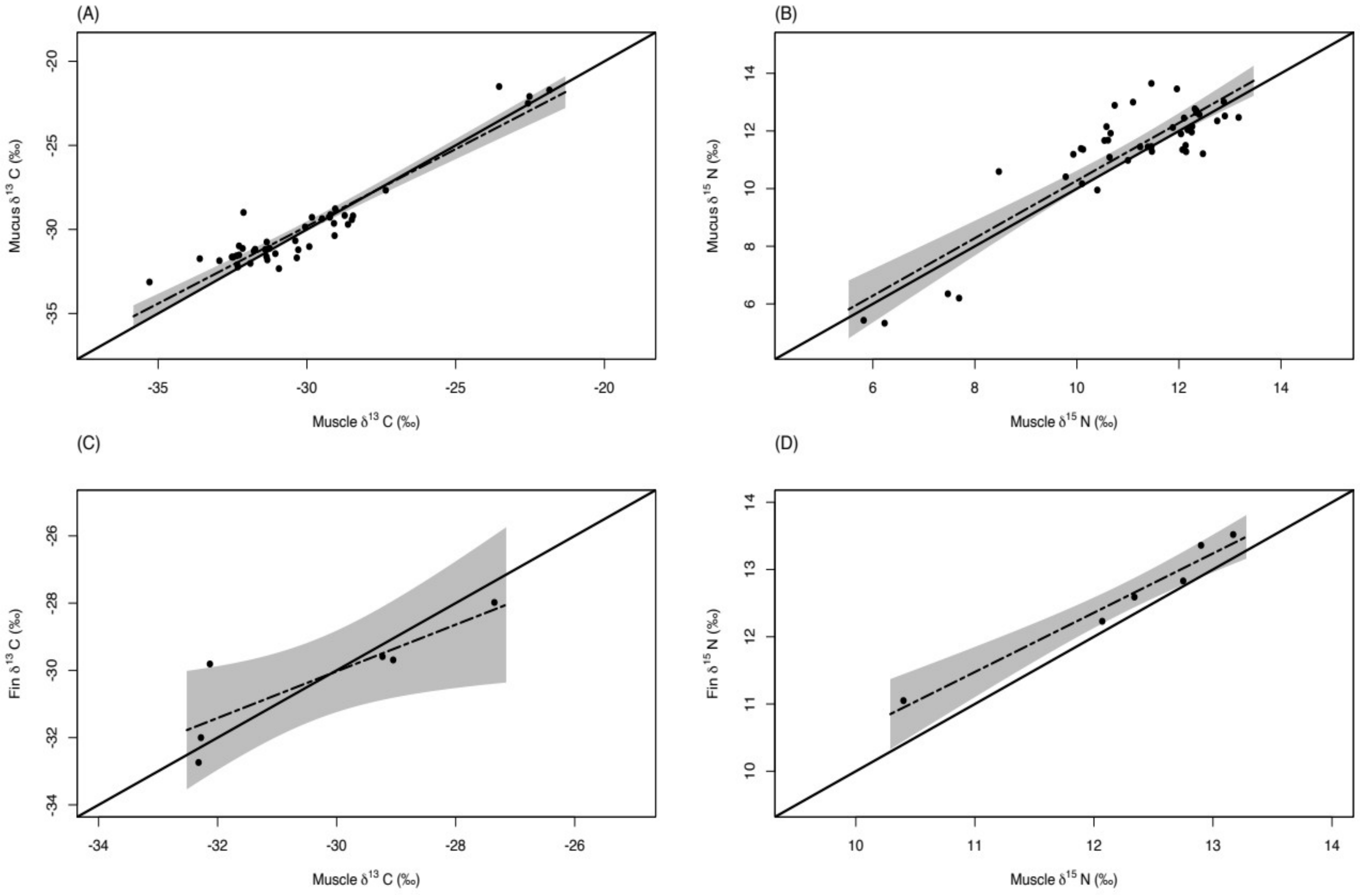

(A)
Mucus δ13 C (‰)
Muscle δ13 C (‰)
(B)
Mucus δ15 N (‰)
Muscle δ15 N (‰)
(C)
Fin δ13 C (‰)
Muscle δ13 C (‰)
(D)
Fin δ15 N (‰)
Muscle δ15 N (‰)
